# Increased cerebrospinal fluid YKL-40 concentration in hip fracture patients with delirium

**DOI:** 10.1093/braincomms/fcag005

**Published:** 2026-02-25

**Authors:** Thea Berntsen, Kaj Blennow, Henrik Zetterberg, Ingrid Fæhn Brekke, Mathias Nikolai Petersen Hella, Tom Tarjei Lian, Lene B Solberg, Christian Thomas Pollmann, Adi Karabeg, Olav Tobias Ødegaard, Marius Myrstad, Kristian Sydnes, Roy Bjørkholt Olsen, Torgeir Bruun Wyller, Leiv Otto Watne, Bjørn Erik Neerland

**Affiliations:** Oslo Delirium Research Group, Institute of Clinical Medicine, University of Oslo, Oslo 0318, Norway; Department of Geriatric Medicine, Oslo University Hospital, Oslo 0424, Norway; Department of Psychiatry and Neurochemistry, Institute of Neuroscience and Physiology, the Sahlgrenska Academy at the University of Gothenburg, Mölndal S-431 80, Sweden; Clinical Neurochemistry Laboratory, Sahlgrenska University Hospital, Mölndal S-431 80, Sweden; Paris Brain Institute, ICM, Pitié-Salpêtrière Hospital, Sorbonne University, Paris 75646, France; Neurodegenerative Disorder Research Center, Division of Life Sciences and Medicine, Department of Neurology, Institute on Aging and Brain Disorders, University of Science and Technology of China and First Affiliated Hospital of USTC, Hefei 230001, China; Department of Psychiatry and Neurochemistry, Institute of Neuroscience and Physiology, the Sahlgrenska Academy at the University of Gothenburg, Mölndal S-431 80, Sweden; Clinical Neurochemistry Laboratory, Sahlgrenska University Hospital, Mölndal S-431 80, Sweden; Department of Neurodegenerative Disease, UCL Institute of Neurology, London WC1E 6BT, UK; UK Dementia Research Institute at UCL, London WC1E 6BT, UK; Hong Kong Center for Neurodegenerative Diseases, Hong Kong Science Park, Shatin, People’s Republic of China; Wisconsin Alzheimer’s Disease Research Center, University of Wisconsin School of Medicine and Public Health, University of Wisconsin-Madison, Madison, WI 53792-2420, USA; Department of Anaesthesiology, Oslo University Hospital, Oslo 0424, Norway; Department of Geriatric Medicine, Stavanger University Hospital, Stavanger 4011, Norway; Department of Clinical Medicine, University of Bergen, Bergen 5021, Norway; Department of Orthopaedic Surgery, Bærum Hospital, Vestre Viken Hospital Trust, Gjettum 1346, Norway; Division of Orthopaedic Surgery, Oslo University Hospital, Oslo 0424, Norway; Department of Orthopaedic Surgery, Akershus University Hospital, Lørenskog 1478, Norway; Department of Orthopaedic Surgery, Akershus University Hospital, Kongsvinger 2212, Norway; Department of Anaesthesiology, Akershus University Hospital, Kongsvinger 2212, Norway; Department of Internal Medicine, Bærum Hospital, Vestre Viken Hospital Trust, Gjettum 1346, Norway; Department of Surgery and Anaesthesia, Unit for Orthopaedic Surgery, Diakonhjemmet Hospital, Oslo 0319, Norway; Department of Anaesthesiology and Intensive Care, Sørlandet Hospital, Arendal 4838, Norway; Oslo Delirium Research Group, Institute of Clinical Medicine, University of Oslo, Oslo 0318, Norway; Department of Geriatric Medicine, Oslo University Hospital, Oslo 0424, Norway; Oslo Delirium Research Group, Institute of Clinical Medicine, University of Oslo, Oslo 0318, Norway; Department of Geriatric Medicine, Akershus University Hospital, Lørenskog 1478, Norway; Oslo Delirium Research Group, Institute of Clinical Medicine, University of Oslo, Oslo 0318, Norway; Department of Geriatric Medicine, Oslo University Hospital, Oslo 0424, Norway

**Keywords:** delirium, YKL-40, neuroinflammation, Alzheimer’s disease, CSF biomarkers

## Abstract

The underlying mechanisms of the neuropsychiatric syndrome delirium are still unknown, but neuroinflammation is a central hypothesis. Chitinase-3-like-protein-1 (YKL-40/*CHI3L1*) is considered a marker of neuroinflammation when measured in cerebrospinal fluid (CSF). The aim of this study was to examine concentrations of CSF YKL-40 in patients with and without delirium, to enhance the understanding of delirium pathophysiology.

A total of 545 hip fracture patients were included from two similar cohorts. CSF samples were collected in conjunction with spinal anaesthesia for hip fracture surgery. The patients were screened for delirium both pre- and postoperatively. Those with delirium were further divided into subgroups based on whether they developed it before surgery (prevalent delirium) or after surgery (incident delirium). Among patients without delirium, those who met some, but not all diagnostic criteria, were classified as having subsyndromal delirium. Prefracture cognitive function was assessed, and American Society of Anaesthesiologists physical status score was included as a marker of comorbidity.

In total, 257 (47%) of the patients developed delirium. These patients were older and had a higher prevalence of dementia and severe systemic diseases. Among the patients *without* dementia, those with delirium had higher median concentration of CSF YKL-40 compared with those without delirium (first cohort: 175 versus 132 ng/mL, *P* = 0.01, second cohort: 243 versus 174 ng/mL, *P* < 0.001). No association was found among the patients *with* dementia. The results remained consistent when adjusting for age and comorbidity. No difference in median CSF YKL-40 concentration was found between patients who had delirium at the time of surgery (prevalent delirium) and those who developed it afterwards (incident delirium).

Our findings support the hypothesis of neuroinflammation as a mechanism for delirium in patients without dementia.

## Teaser

Berntsen *et al*. investigated the relationship between delirium and the protein YKL-40 in cerebrospinal fluid. They found that concentrations of YKL-40 were increased in delirious hip fracture patients without dementia. This finding supports the hypothesis of neuroinflammation as a mechanism for delirium in these patients.

## Graphical Abstract

**Graphical Abstract:**
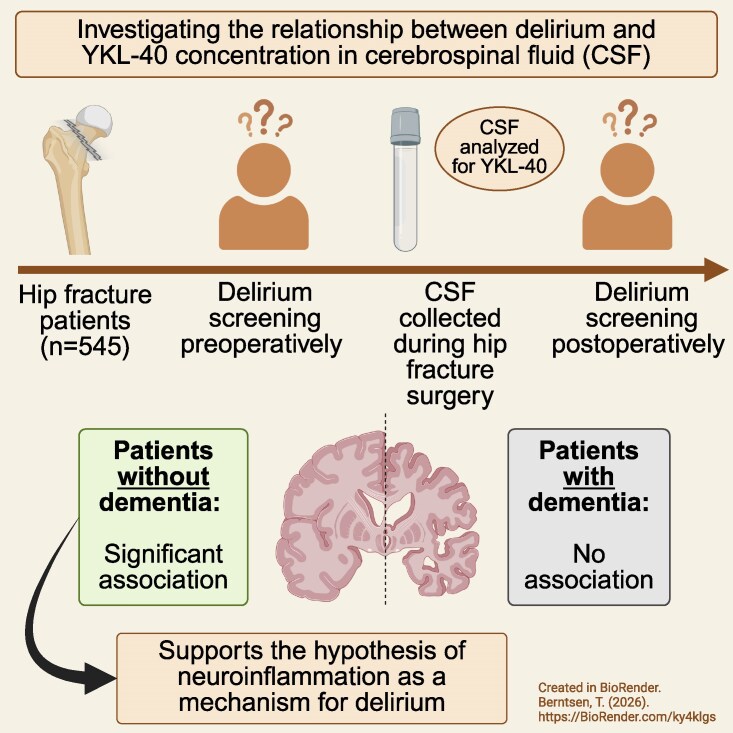

## Introduction

Delirium is a severe and common syndrome characterized by acute onset of altered awareness and disrupted attention.^1^ Among patients with hip fractures, delirium occurs in up to 50%,^2^ with old age, dementia and other comorbidities being important predisposing factors and trauma and surgery being precipitating factors.^3^

While significant advancements have been made in understanding the epidemiology of delirium and detecting it using screening tools, uncertainties surrounding delirium pathophysiology persist.^1,3^ Due to the variety of different aetiologies leading to delirium, it has been proposed that several different mechanisms might be involved. Common hypotheses include neuroinflammation,^4^ altered cerebral metabolism^5^ and neurotransmitter imbalance.^6^ The neuroinflammation hypothesis encompasses induction of systemic inflammatory pathways with release of inflammatory mediators, glial cell activation and disruption of the blood–brain barrier.^1,4^

Researching blood and cerebrospinal fluid (CSF) biomarkers of delirium has become important for enhancing our understanding of its underlying mechanisms^7^ and potentially enabling targeted treatment options.^8^ Several biomarkers have so far been linked to delirium, including neurotransmitters, hormones, indicators of neuronal damage and markers of inflammation.^7,8^

YKL-40, also known as chitinase-3-like-protein-1 (encoded by *CHI3L1*), is a glycoprotein expressed by several different cell types such as macrophages, neutrophils, tumour cells and glial cells.^9,10^ The protein has several functions, such as the activation of immune cells and promotion of tissue remodelling.^11^ Human studies have demonstrated that concentrations of YKL-40 in blood are elevated in various inflammatory diseases.^9^ Measured in CSF, the protein is regarded a nonspecific marker of neuroinflammation, reflecting glial cell activation (both microglia and astrocytes).^12,13^ Elevated CSF YKL-40 concentration is associated with acute neuroinflammatory disorders, i.e. CNS infections,^14^ and neurodegenerative disorders such as multiple sclerosis^15^ and Alzheimer’s disease.^16^ YKL-40 concentration has also been reported to increase with age, both in blood and CSF.^9,17,18^

Although dementia and delirium are two distinct conditions, they share some clinical features.^19^ Moreover, similar to Alzheimer’s disease, growing evidence suggests that neuroinflammation and glial cell involvement also play a role in the pathophysiology of delirium.^20^ Despite this, the relationship between YKL-40 and delirium is sparsely investigated. In 2021, Vasunilashorn *et al.*^21^ conducted a proteome-wide analysis of plasma collected from older patients undergoing major elective surgery. They found that elevated YKL-40 concentrations in both preoperative and postoperative blood samples were associated with postoperative delirium, suggesting YKL-40 as a promising delirium biomarker. However, their study involved a relatively healthy population without dementia, limiting the generalizability. Furthermore, blood biomarkers primarily reflect peripheral processes, while CSF biomarkers are thought to better represent pathological processes in the brain.^22^ To our knowledge, only David-Bercholz *et al.*^23^ have investigated YKL-40 concentration in CSF in delirious patients. They analysed CSF samples from older patients before and after non-neurologic, non-cardiac surgery and found no differences in post-surgical changes of YKL-40 concentration between patients with and without postoperative delirium. However, their study included only 22 participants.

Hence, the aim of our study was to further investigate the relationship between CSF YKL-40 and delirium and thus advance our understanding of potential neuroinflammatory mechanisms underlying delirium. We included patients with hip fracture because of the high prevalence of delirium and dementia in this patient group, along with the opportunity to collect CSF both prior to surgery and, for some patients, during ongoing delirium. Based on findings from previous studies on neuroinflammatory and neurodegenerative disorders, we hypothesized that CSF YKL-40 concentration would be higher in those with delirium than in those without.

## Materials and methods

### Participants

**Figure 1:**
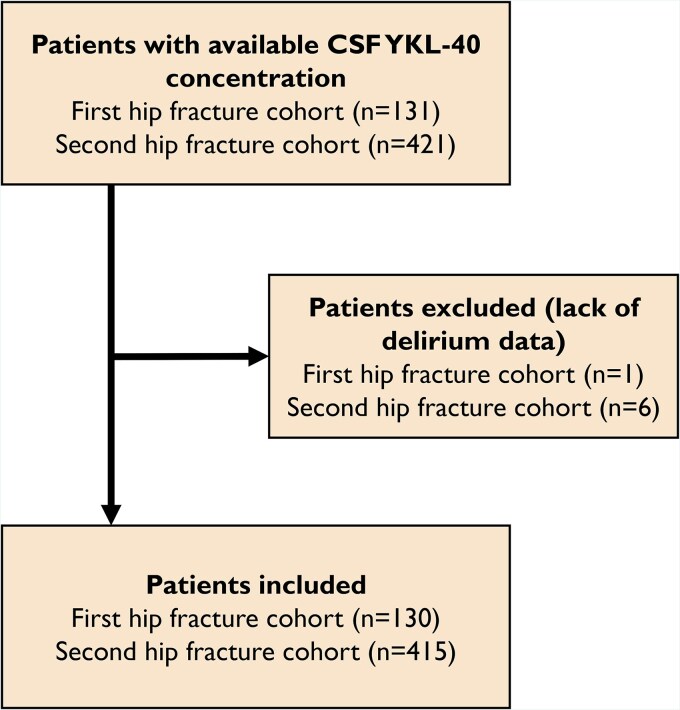
**Flowchart of patient selection**.

Patients with available CSF YKL-40 concentration were recruited from two prospective hip fracture cohorts (Fig. 1). In these cohorts, all patients admitted with hip fracture, undergoing surgery with spinal anaesthesia, were considered for inclusion. The first cohort consists of patients included between 2009 and 2012, originally participating in the Oslo Orthogeriatric Trial at Oslo University Hospital, Norway.^2^ Patients with high energy trauma or terminal illness were excluded. The second cohort consists of patients included between 2016 and 2020 at five different hospitals in Eastern Norway (Diakonhjemmet Hospital, Oslo University Hospital, Baerum Hospital, Akershus University Hospital and Kongsvinger Hospital). The only exclusion criterion in this cohort was inability to obtain CSF. Among those with available CSF YKL-40 concentration, seven were excluded due to missing delirium status, resulting in a final sample of 545 hip fracture patients.

### Delirium assessments

In both hip fracture cohorts, trained personnel screened all patients for delirium preoperatively and until the fifth postoperative day. Patients with delirium were screened until discharge. In the first cohort, delirium was assessed using the Confusion Assessment Method (CAM),^24^ as previously described.^2,25^ Subsyndromal delirium (SSD) was defined as having two or more positive CAM items, without meeting the full criteria for delirium. In the second cohort, delirium was assessed according to the Diagnostic and Statistical Manual of Mental Disorders-5 (DSM-5) criteria,^26^ by a standardized procedure described earlier.^27,28^ For patients not fulfilling these criteria, SSD was defined as an acute change in mental status, combined with either (i) altered arousal; (ii) attentional deficits; (iii) other cognitive changes; or (iv) delusions or hallucinations. Both methods of delirium assessments were based on interviews with the patients as well as information from relatives, nurses and hospital records. Prefracture cognitive function was factored into the final diagnosis. Delirium was classified as a binary variable (delirium/no delirium) and further divided into subgroups. Those with delirium were categorized as having either prevalent (onset before surgery) or incident (onset after surgery) delirium. Those without delirium were categorized as having either SSD or never delirium.

### Assessment of prefracture cognitive function and comorbidity

In the first hip fracture cohort, one geriatrician and one old age psychiatrist independently evaluated whether the patients met the International Classification of Diseases 10th revision (ICD-10) criteria for dementia,^29^ based on all existing clinical data except for delirium status. Proxies were interviewed with the Informant Questionnaire on Cognitive Decline in the Elderly (IQCODE),^30^ the Barthel Activities of Daily Living (ADL) index^31^ and the Nottingham Extended ADL index.^32^ In the second cohort, dementia was defined as an IQCODE average score ≥ 3.44. When IQCODE was missing (*n* = 36), hospital records were used to determine dementia status retrospectively. The American Society of Anaesthesiologists classification of physical status^33^ (ASA score) was collected from hospital records in both cohorts, as a marker of comorbidity. For this study, patients were classified as having either ASA score I–II or III–IV.

### CSF sampling and handling

#### CSF collection and processing

In both cohorts, up to 5 mL of CSF was collected immediately prior to spinal anaesthesia. Within 4 h after collection, the CSF sample was centrifuged at 2000 *g* for 10 min in room temperature, aliquoted and stored at −80°C. Samples were transported on dry ice and analysed at the Clinical Neurochemistry Laboratory at Sahlgrenska University Hospital (Mölndal, Sweden).

#### CSF YKL-40 measurement

Samples from the first hip fracture cohort were analysed for YKL-40 in 2017, while samples from the second hip fracture cohort were analysed for YKL-40 in 2021. On both occasions, YKL-40 concentration was measured using a commercial Enzyme Linked Immunosorbent Assay (R&D Systems, Minneapolis, MN). The measurements were performed by board-certified laboratory technicians who were blind to the clinical data.

### Ethics

Written informed consent was obtained from all study participants or from their proxies if the participants were unable to provide consent. The study complied with the Declaration of Helsinki and was approved by the Regional Committee for Ethics in Medical Research in Norway (REK 2009/450, REK 2016/1368).

### Statistical analysis

Although the same assay was used for analysing CSF YKL-40 in 2017 and 2021, changes in the absolute concentrations across kit lots cannot be completely excluded. Therefore, the results from the first and second hip fracture cohort were not directly comparable, and univariable analyses were conducted separately for the two cohorts. Patients with delirium were compared to patients without delirium. Because the distribution of age and YKL-40 concentrations was skewed, non-parametric tests were used for continuous data. Mann–Whitney U-test was used for comparing the median value of age and YKL-40 concentration between the groups. Chi-square test was used for comparing categorical data (sex, dementia status and ASA score) between the groups. The tests were repeated after stratifying the cohorts by dementia status. Kruskal–Wallis test was used for analysing differences in median YKL-40 concentration between subgroups (prevalent delirium, incident delirium, SSD and never delirium) and repeated after stratifying the cohorts by dementia status. When a significant difference between subgroups were identified, Dunn’s test with Bonferroni adjustment was used for pairwise comparisons of subgroups, in order to determine which specific subgroups differed from each other. The statistical tests were two tailed, and the significance level was set at 0.05.

For multivariable analysis, we performed logistic regression with delirium as the outcome variable. To increase statistical power, we merged the two hip fracture cohorts and adjusted for the systematic differences in CFS YKL-40 concentrations across cohorts by adding a variable representing each cohort. Other covariates were added in the model using a stepwise procedure including all variables from the univariable analyses (age, sex, dementia status and ASA score). All, except for sex, were considered confounding factors and included in the final model. We assessed for linearity using Box–Tidwell test, and multicollinearity using variance inflation factor and a correlation matrix. The adjusted model was then evaluated for potential effect modification by adding interaction terms in the model. All possible interactions between the covariates were examined. We identified a significant interaction between dementia and CSF YKL-40 (*P* < 0.001), which altered the association between delirium and YKL-40 from non-significant (*P* = 0.870) to significant (*P* = 0.002). To explore the impact of this interaction further, we stratified the regression model by dementia status. We assessed for influential observations in both models, and those with the highest impact on the model estimates were excluded. See the Supplementary material for details regarding influential observations.

All statistical analyses and graphs were performed using StataCorp. 2023. Stata Statistical Software: Release 18. College Station, TX: StataCorp LLC.

## Results

### Demographics

Demographics of the two hip fracture cohorts were similar (Table 1). In total, 257 (47%) of the participants had delirium, 225 (41%) had dementia and 290 (53%) had an ASA score of III–IV. The median age was 83 years, with a range from 32 to 101 years, and most of the participants were female (*n* = 372, 68%). Those with delirium were older and had a higher prevalence of dementia and severe systemic diseases (ASA score III–IV) compared with those without delirium. Patient sex was not associated with delirium.

**Table 1:** Characteristics and CSF YKL-40 concentrations of the participants in both hip fracture cohorts, according to delirium status

	All (*n* = 545)	Delirium (*n* = 257)	No delirium (*n* = 288)	*P*-value^a^
**First hip fracture cohort, *n***	**130**	**69**	**61**	
Age, median (IQR)	85 (80–89)	85 (81–89)	84 (73–88)	**0**.**020**
Female, *n* (%)	94 (72)	47 (68)	47 (77)	0.256
Dementia, *n* (%)**^b^**	62 (48)	52 (75)	10 (16)	**<0**.**001**
ASA score III–IV, *n* (%)	76 (58)	49 (71)	27 (44)	**0**.**002**
CSF YKL-40, ng/mL, median (IQR)**^c^**	157 (115–204)	168 (120–224)	138 (102–198)	**0**.**032**
**Second hip fracture cohort, *n***	**415**	**188**	**227**	
Age, median (IQR)	82 (74–88)	86 (80–91)	77 (70–85)	**<0**.**001**
Female, *n* (%)	278 (67)	127 (68)	151 (67)	0.820
Dementia, *n* (%)**^d^**	163 (39)	131 (70)	32 (14)	**<0**.**001**
ASA score III–IV, *n* (%)**^e^**	214 (52)	127 (68)	87 (38)	**<0**.**001**
CSF YKL-40, ng/mL, median (IQR)**^c^**	200 (155–279)	226 (179–314)	180 (141–240)	**<0**.**001**

IQR, interquartile range (25th percentile–75th percentile); ASA, American Society of Anaesthesiologists physical status.

^a^Hip fracture patients with delirium versus without delirium. Mann–Whitney U-test was used for continuous variables, and chi-square test for categorical variables. Bold text denotes a significant *P*-value (*P* < 0.05).

^b^Expert consensus diagnosis.

^c^CSF YKL-40 concentrations are not directly comparable between cohorts.

^d^IQCODE average score ≥ 3.44, expert consensus diagnosis where IQCODE was missing (*n* = 36).

^e^ASA score was missing in two participants.

### CSF YKL-40 and delirium

**Figure 2:**
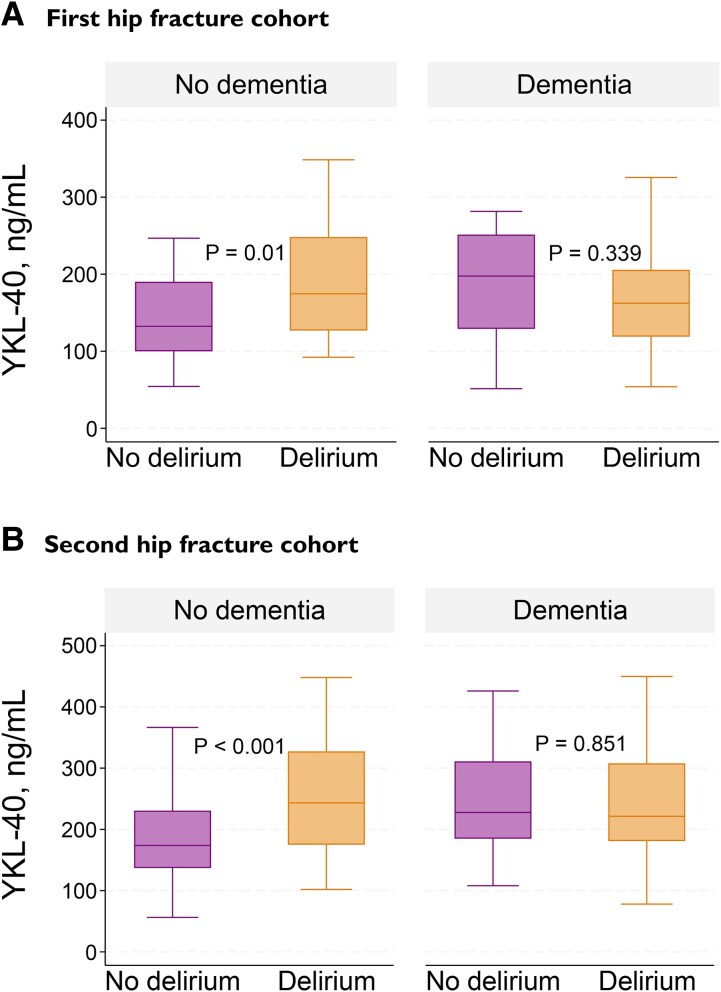
**CSF YKL-40 concentration in patients with and without delirium, stratified by dementia status**. Mann–Whitney U-test was used for comparing median CSF YKL-40 concentration between the groups. (**A**) First hip fracture cohort: in the no dementia group, patients with delirium (*n* = 17) had significantly higher median CSF YKL-40 concentration than those without delirium (*n* = 51). In the dementia group, there was no significant difference between the patients with (*n* = 52) and without (*n* = 10) delirium. (**B**) Second hip fracture cohort: in the no dementia group, patients with delirium (*n* = 57) had significantly higher median CSF YKL-40 concentration than those without delirium (*n* = 195). In the dementia group, there was no significant difference between the patients with (*n* = 131) and without (*n* = 32) delirium. The centre line in the boxes represents the median (50th percentile). The bottom and top of the boxes are the 25th and 75th percentiles. The ‘whiskers’ extend to the minimum and maximum values within 1.5 times the difference between the 25th percentile and the 75th percentile. Outliers beyond this range were excluded for visual purposes but included in the statistical analyses.

In both cohorts, patients with delirium had significantly higher median CSF YKL-40 concentration compared with those without delirium (Table 1). When stratifying the cohorts according to dementia status, this difference was only observed for patients without dementia (first cohort: *n* = 68, 175 versus 132 ng/mL, *P* = 0.01, second cohort: *n* = 252, 243 versus 174 ng/mL, *P* < 0.001) (Fig. 2). A full table with results from the stratified analyses is provided in the Supplementary material (Supplementary Table 1).

**Figure 3:**
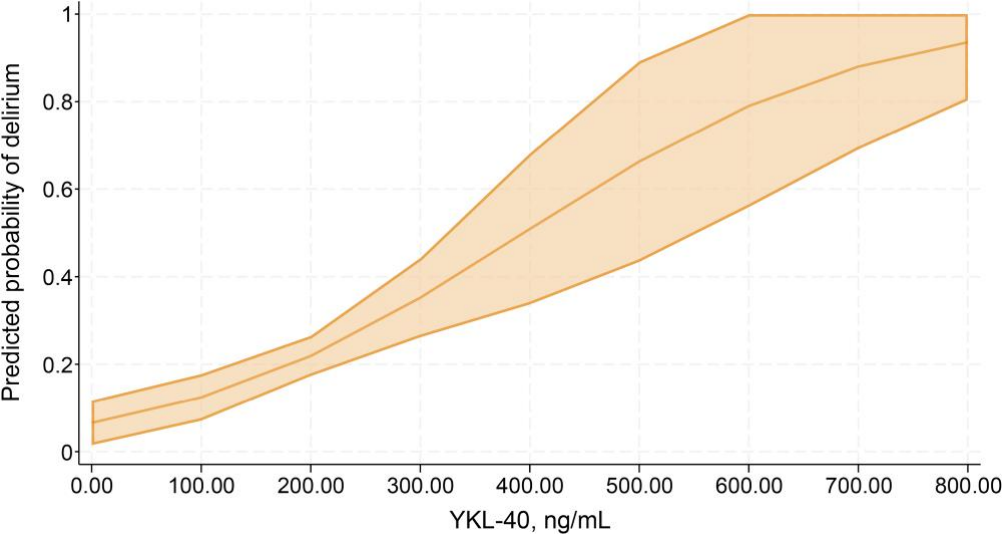
**Predicted probabilities of delirium for different YKL-40 concentrations for patients without dementia, when age and ASA score are at their mean values.** The graph is a margins plot calculated from a logistic regression model including patients without dementia (*n* = 318), with delirium as the outcome variable and CSF YKL-40, age, ASA score and a cohort variable as covariates. CSF YKL-40 was significantly associated with delirium (OR 1.007, 95% CI 1.004–1.011, *P* > 0.001). The shaded area represents the 95% confidence interval for the predicted probabilities. One observation was excluded from the logistic regression model because of missing ASA score and one because it was considered an influential observation.

In multivariable analysis, where we combined the two hip fracture cohorts, adjusting for age and ASA score in stratified models did not alter the results (Table 2; Fig. 3).

**Table 2:** Multivariable logistic regression with delirium as outcome, stratified by dementia status

	Adjusted OR	95% CI	*P*-value^a^
**Patients without dementia (*n* = 318^b^)**			
CSF YKL-40, ng/mL	1.007	1.004–1.011	**<0**.**001**
Age	1.049	1.013–1.087	**0**.**007**
ASA score III–IV	2.122	1.179–3.819	**0**.**012**
**Patients with dementia (*n* = 222^c^)**			
CSF YKL-40, ng/mL	0.997	0.993–1.001	0.189
Age	1.09	1.039–1.143	**<0**.**001**
ASA score III–IV	2.412	1.164–4.998	**0**.**018**

A cohort variable was also included in both models to adjust for differences in YKL-40 concentration between cohorts, but is not shown in this table due to its lack of clinical relevance.

ASA, American Society of Anaesthesiologists physical status.

^a^Bold text denotes a significant *P*-value (*P* < 0.05).

^b^One observation excluded because of missing ASA score and one because it was an influential observation.

^c^One observation excluded because of missing ASA score and two because they were influential observations.

### CSF YKL-40 and delirium subgroups

**Figure 4:**
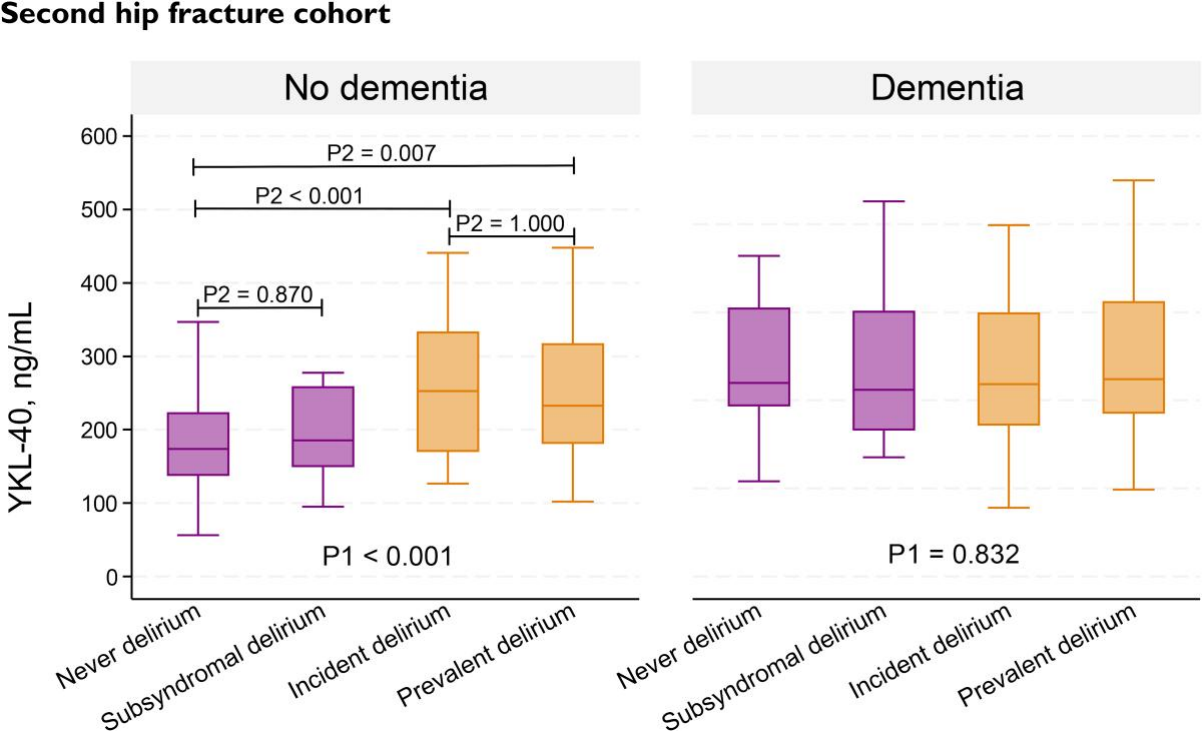
**Comparison of CSF YKL-40 concentration in delirium subgroups in the second hip fracture cohort, stratified by dementia status.**  *P1* = *P*-values from Kruskal–Wallis test (overall comparison of all four subgroups). *P2* = *P*-values from Dunn’s test after Bonferroni adjustment (pairwise comparison of the individual subgroups). The endpoints of the horizontal lines indicate which two subgroups are being compared with each other. In the no dementia group (*n* = 252), median CSF YKL-40 concentration was significantly higher in the incident (*n* = 37) and prevalent (*n* = 20) delirium subgroups, compared to the never delirium group (*n* = 177). There was no significant difference between the incident and prevalent subgroups, or between the never and subsyndromal (*n* = 18) subgroups. In the dementia group (*n* = 163), no difference between the subgroups were identified (never delirium, *n* = 19; SSD, *n* = 12; incident delirium, *n* = 59; prevalent delirium, *n* = 70; and missing, *n* = 3). The centre line in the boxes represents the median (50th percentile). The bottom and top of the boxes are the 25th and 75th percentiles. The ‘whiskers’ extend to the minimum and maximum values within 1.5 times the difference between the 25th percentile and the 75th percentile. Outliers beyond this range were excluded for visual purposes but included in the statistical analyses.

Among the patients with delirium, a total of 133 (52%) had prevalent delirium (first cohort: *n* = 43, second cohort: *n* = 90), and 120 (47%) had incident delirium (first cohort: *n* = 24, second cohort: *n* = 96). Among those without delirium, a total of 52 (18%) had SSD (first cohort: *n* = 22, second cohort: *n* = 30). Subgroup status was missing for four patients with delirium and for one patient without delirium. We were only able to detect significant differences in median CSF YKL-40 concentration between the four subgroups in the second hip fracture cohort (Fig. 4). Consistent with the previous analyses, these differences were only observed for the patients without dementia. When comparing the subgroups within this group, YKL-40 concentration in both the prevalent and incident delirium subgroups differed significantly from the never delirium subgroup. There were no significant differences in YKL-40 concentration between the incident and prevalent subgroups or between the never delirium and subsyndromal subgroups.

## Discussion

The main finding of this study was that CSF YKL-40 concentration was significantly associated with delirium in hip fracture patients *without* dementia. Higher CSF YKL-40 concentration was associated with increased probability of delirium, also when adjusting for age and comorbidity (ASA score). Among the patients *with* dementia, no significant association between CSF YKL-40 concentration and delirium was found. Our findings suggest that YKL-40 could be involved in delirium pathophysiology, strengthening the hypothesis of neuroinflammation and providing insight to the link between hip fracture and delirium.

There could be various explanations for why the association between CSF YKL-40 and delirium was dependent on prefracture dementia status. Firstly, we are aware that diagnosing delirium superimposed on dementia is challenging.^34^ Hence, methodological issues among the patients with dementia may influence our results. However, other studies investigating associations between inflammatory biomarkers and delirium have found similar results, supporting a biological explanation.

McNeil *et al.*,^35^ investigating delirium in patients admitted to an emergency department, found that blood interleukin-6, a marker of peripheral inflammation, was associated with delirium duration only in patients without dementia. They suggested that people with dementia, being more susceptible to delirium, may only require a small inflammatory stimulus to become delirious. Thus, differences in inflammatory markers in these patients may be too small to detect. Neerland *et al.*,^36^ investigating patients from our first hip fracture cohort along with hip fracture patients from Edinburgh, found that C-reactive protein concentration in CSF was associated with delirium only among the patients without dementia. Henjum *et al*.,^37^ also investigating patients from our first hip fracture cohort, found a similar relationship for the soluble triggering receptor expressed on myeloid cell 2 (sTREM2) in CSF. sTREM2 is considered a marker of microglial activation and has been shown to correlate positively with CSF YKL-40.^38^ Henjum *et al.*^37^ speculated that microglia are continuously stimulated in patients with clinical Alzheimer’s disease, resulting in reduced microglial response and subsequently lower release of sTREM2 in these patients during delirium.

Several studies have demonstrated that CSF YKL-40 concentration rises early in the Alzheimer’s disease continuum, indicating that activation of microglia and astrocytes occurs in the early stages of Alzheimer’s disease.^39,40^ However, few longitudinal studies examining CSF YKL-40 concentration across different stages of dementia exist. Wennström *et al*.^18^ suggested that CSF YKL-40 concentration might reach a plateau, or even decline, in the later stages of Alzheimer’s disease. Thus, we speculate that patients with advanced dementia, especially Alzheimer’s disease, may be less capable of increased glial cell activation during a peripheral inflammatory response, such as in hip fractures, and that other mechanisms may be more important in the development of delirium in these patients.

We found no differences in CSF YKL-40 concentration between patients who exhibited delirium at the time of CSF sampling (prevalent delirium) and those who developed delirium afterwards (incident delirium), regardless of dementia status. This could indicate that the extent of glial cell activation, marked by YKL-40, remains consistent both prior to and following the onset of delirium.

### Strengths and limitations

To our knowledge, this is one of the largest CSF studies within delirium research. As we aimed to enhance the understanding of delirium pathophysiology, particularly looking at a neurological mechanism, the use of CSF is an important strength. Additionally, while the patient group was heterogeneous and several factors may have contributed to the development of delirium, the patients shared a common primary aetiology (hip fracture), which enhances the likelihood of shared pathological mechanisms among them. Furthermore, with minimal exclusion criteria, we believe our findings are relevant to most hip fracture patients.

Although different diagnostic methods for delirium were used for the two hip fracture cohorts, we consider both methods as valid and reliable. The consistent results observed in both cohorts further support this. Including data on prefracture dementia is another strength of the study. Dementia acts as a confounding variable, influencing both delirium and the YKL-40 concentration, and as an effect modifier, affecting how YKL-40 impacts delirium. However, while IQCODE is validated for diagnosing dementia in delirious patients,^(41)^ relying solely on IQCODE, as we did for the second cohort, presents a limitation.

Since we only collected CSF samples at a single time point, we were unable to assess changes in YKL-40 concentration in the same patient over time, which could have given us a better understanding of the neuroinflammatory trajectory in hip fracture patients. However, obtaining multiple CSF samples from the same patient, at different times, poses practical and ethical challenges. Registering whether the patients experienced prevalent or incident delirium was an alternative approach to provide similar insights. Still, by using this approach, we lose some statistical power.

Finally, not accounting for medication use is an important limitation. Pre-admission use of anti-inflammatory drugs may influence CSF YKL-40 concentration, while medications like opioids and benzodiazepines could impact the occurrence of delirium.^1^

## Conclusion

Our findings indicate that increased CSF concentration of the protein YKL-40 is associated with delirium in hip fracture patients without dementia, strengthening the hypothesis of neuroinflammation as a mechanism for delirium in these patients. For patients with dementia, other mechanisms may be of greater importance. Future studies investigating inflammatory biomarkers of delirium should include patients both with and without prefracture dementia, to examine this relationship further. Although challenging, future studies should also aim to collect CSF samples at multiple time points to enable stronger interpretations of underlying mechanisms.

## Supplementary Material

fcag005_Supplementary_Data

## Data Availability

Access to the dataset is restricted for ethical reasons and available only upon request, which can be directed to theabern@uio.no. Those requesting data will be required to sign a data access agreement. Statistical analyses were conducted by using standard codes in Stata 18. The following packages were installed: boxtid (Box–Tidwell test), collin (collinearity diagnostics), and st0381_1 (Dunn’s test). No new codes were generated for this study.

## References

[1] Wilson JE, Mart M, Cunningham C, et al Delirium. Nat Rev Dis Primer. 2020;6(1):90. 10.1038/s41572-020-00223-4 PMC9012267 33184265

[2] Watne LO, Torbergsen AC, Conroy S, et al The effect of a pre- and postoperative orthogeriatric service on cognitive function in patients with hip fracture: Randomized controlled trial (Oslo Orthogeriatric Trial). BMC Med. 2014;12(1):63. 24735588 10.1186/1741-7015-12-63 PMC4022270

[3] Inouye SK, Westendorp RG, Saczynski JS. Delirium in elderly people. The Lancet. 2014;383(9920):911–922. 10.1016/S0140-6736(13)60688-1 PMC4120864 23992774

[4] Cerejeira J, Firmino H, Vaz-Serra A, Mukaetova-Ladinska EB. The neuroinflammatory hypothesis of delirium. Acta Neuropathol. 2010;119(6):737–754. 20309566 10.1007/s00401-010-0674-1

[5] Titlestad I, Watne LO, Caplan GA, et al Impaired glucose utilization in the brain of patients with delirium following hip fracture. Brain. 2024;147(1):215–223. 37658825 10.1093/brain/awad296 PMC10766236

[6] Maldonado JR . Delirium pathophysiology: An updated hypothesis of the etiology of acute brain failure. Int J Geriatr Psychiatry. 2018;33(11):1428–1457. 29278283 10.1002/gps.4823

[7] Lozano-Vicario L, García-Hermoso A, Cedeno-Veloz BA, et al Biomarkers of delirium risk in older adults: A systematic review and meta-analysis. Front Aging Neurosci. 2023;15:1174644. 37251808 10.3389/fnagi.2023.1174644 PMC10213257

[8] Smith CJ, Hodge D, Harrison FE, Roberson SW. The pathophysiology and biomarkers of delirium. Semin Neurol. 2024;44(6):720–731. 39419070 10.1055/s-0044-1791666 PMC11622424

[9] Blazevic N, Rogic D, Pelajic S, et al YKL-40 as a biomarker in various inflammatory diseases: A review. Biochem Medica. 2024;34(1):010502. 10.11613/BM.2024.010502 PMC10731731 38125621

[10] Bonneh-Barkay D, Wang G, Starkey A, Hamilton RL, Wiley CA. In vivo CHI3L1 (YKL-40) expression in astrocytes in acute and chronic neurological diseases. J Neuroinflammation. 2010;7(1):34. 20540736 10.1186/1742-2094-7-34 PMC2892443

[11] Connolly K, Lehoux M, O’Rourke R, et al Potential role of chitinase-3-like protein 1 (CHI3L1/YKL-40) in neurodegeneration and Alzheimer’s disease. Alzheimers Dement. 2023;19(1):9–24. 35234337 10.1002/alz.12612 PMC9437141

[12] Baldacci F, Lista S, Palermo G, Giorgi FS, Vergallo A, Hampel H. The neuroinflammatory biomarker YKL-40 for neurodegenerative diseases: Advances in development. Expert Rev Proteomics. 2019;16(7):593–600. 31195846 10.1080/14789450.2019.1628643

[13] Llorens F, Thüne K, Tahir W, et al YKL-40 in the brain and cerebrospinal fluid of neurodegenerative dementias. Mol Neurodegener. 2017;12(1):83. 29126445 10.1186/s13024-017-0226-4 PMC5681777

[14] Østergaard C, Johansen JS, Benfield T, Price PA, Lundgren JD. YKL-40 is elevated in cerebrospinal fluid from patients with purulent meningitis. Clin Vaccine Immunol. 2002;9(3):598–604. 10.1128/CDLI.9.3.598-604.2002 PMC119997 11986266

[15] Floro S, Carandini T, Pietroboni AM, De Riz MA, Scarpini E, Galimberti D. Role of chitinase 3–like 1 as a biomarker in multiple sclerosis. Neurol Neuroimmunol Neuroinflammation. 2022;9(4):e1164. 10.1212/NXI.0000000000001164 PMC9128043 35534236

[16] Alzheimer’s Disease vs Control: YKL-40 (CSF) ALZFORUM. Accessed 23 2025. https://www.alzforum.org/alzbiomarker/meta-analysis/alzheimers-disease-vs-control-ykl-40-csf

[17] Craig-Schapiro R, Perrin RJ, Roe CM, et al YKL-40: A novel prognostic fluid biomarker for preclinical Alzheimer’s disease. Biol Psychiatry. 2010;68(10):903–912. 21035623 10.1016/j.biopsych.2010.08.025 PMC3011944

[18] Wennström M, Surova Y, Hall S, et al The inflammatory marker YKL-40 is elevated in cerebrospinal fluid from patients with Alzheimer’s but not Parkinson’s disease or dementia with Lewy bodies. PLoS One. 2015;10(8):e0135458. 26270969 10.1371/journal.pone.0135458 PMC4536228

[19] Fong TG, Inouye SK. The inter-relationship between delirium and dementia: The importance of delirium prevention. Nat Rev Neurol. 2022;18(10):579–596. 36028563 10.1038/s41582-022-00698-7 PMC9415264

[20] Heffernan ÁB, Steinruecke M, Dempsey G, et al Role of glia in delirium: Proposed mechanisms and translational implications. Mol Psychiatry. 2025;30(3):1138–1147. 39463449 10.1038/s41380-024-02801-4 PMC11835730

[21] Vasunilashorn SM, Dillon ST, Chan NY, et al Proteome-wide analysis using SOMAscan identifies and validates chitinase-3-like protein 1 as a risk and disease marker of delirium among older adults undergoing major elective surgery. J Gerontol A Biol Sci Med Sci. 2021;77(3):484–493. 10.1093/gerona/glaa326 PMC8893174 35239952

[22] Blennow K, Zetterberg H. Biomarkers for Alzheimer’s disease: Current status and prospects for the future. J Intern Med. 2018;284(6):643–663. 30051512 10.1111/joim.12816

[23] David-Bercholz J, Acker L, Caceres AI, et al Conserved YKL-40 changes in mice and humans after postoperative delirium. Brain Behav Immun—Health. 2022;26:100555. 36457825 10.1016/j.bbih.2022.100555 PMC9706140

[24] Inouye SK . Clarifying confusion: The confusion assessment method: A new method for detection of delirium. Ann Intern Med. 1990;113(12):941. 2240918 10.7326/0003-4819-113-12-941

[25] Neerland BE, Halaas NB, Idland AV, et al Fatty acid-binding protein 3 in cerebrospinal fluid of hip fracture patients with delirium. J Alzheimers Dis. 2020;77(1):183–190. 32804136 10.3233/JAD-200364

[26] American Psychiatric Association . Diagnostic and statistical manual of mental disorders: DSM-5-TR. 5th ed. American Psychiatric Association Publishing; 2013.

[27] Neerland BE, Hov KR, Bruun Wyller V, et al The protocol of the Oslo study of clonidine in elderly patients with delirium; LUCID: A randomised placebo-controlled trial. BMC Geriatr. 2015;15(1):7. 25887557 10.1186/s12877-015-0006-3 PMC4336683

[28] Pollmann CT, Mellingsæter MR, Neerland BE, Straume-Næsheim T, Årøen A, Watne LO. Orthogeriatric co-management reduces incidence of delirium in hip fracture patients. Osteoporos Int. 2021;32(11):2225–2233. 33963884 10.1007/s00198-021-05974-8 PMC8563591

[29] World Health Organization . The ICD-10 classification of mental and behavioural disorders: Diagnostic criteria for research. World Health Organization; 1993.

[30] Jorm AF . The informant questionnaire on cognitive decline in the elderly (IQCODE): A review. Int Psychogeriatr. 2004;16(3):275–293. 15559753 10.1017/s1041610204000390

[31] Mahoney FI, Barthel DW. Functional evaluation: The Barthel index. Md State Med J. 1965;14:61–65. 14258950

[32] Nouri F, Lincoln N. An extended activities of daily living scale for stroke patients. Clin Rehabil. 1987;1(4):301–305.

[33] Mayhew D, Mendonca V, Murthy BVS. A review of ASA physical status—Historical perspectives and modern developments. Anaesthesia. 2019;74(3):373–379. 30648259 10.1111/anae.14569

[34] Morandi A, Davis D, Bellelli G, et al The diagnosis of delirium superimposed on dementia: An emerging challenge. J Am Med Dir Assoc. 2017;18(1):12–18. 27650668 10.1016/j.jamda.2016.07.014 PMC5373084

[35] McNeil JB, Hughes CG, Girard T, et al Plasma biomarkers of inflammation, coagulation, and brain injury as predictors of delirium duration in older hospitalized patients. PLoS One. 2019;14(12):e0226412. 31856187 10.1371/journal.pone.0226412 PMC6922408

[36] Neerland BE, Hall RJ, Seljeflot I, et al Associations between delirium and preoperative cerebrospinal fluid C-reactive protein, interleukin-6, and interleukin-6 receptor in individuals with acute hip fracture. J Am Geriatr Soc. 2016;64(7):1456–1463. 27341529 10.1111/jgs.14238

[37] Henjum K, Quist-Paulsen E, Zetterberg H, Blennow K, Nilsson LNG, Watne LO. CSF sTREM2 in delirium—Relation to Alzheimer’s disease CSF biomarkers Aβ42, t-tau and p-tau. J Neuroinflammation. 2018;15(1):304. 30390679 10.1186/s12974-018-1331-1 PMC6215363

[38] Heslegrave A, Heywood W, Paterson R, et al Increased cerebrospinal fluid soluble TREM2 concentration in Alzheimer’s disease. Mol Neurodegener. 2016;11(1):3. 26754172 10.1186/s13024-016-0071-x PMC4709982

[39] Janelidze S, Mattsson N, Stomrud E, et al CSF biomarkers of neuroinflammation and cerebrovascular dysfunction in early Alzheimer disease. Neurology. 2018;91(9). 10.1212/WNL.0000000000006082 PMC6133624 30054439

[40] Pelkmans W, Shekari M, Brugulat‐Serrat A, et al Astrocyte biomarkers GFAP and YKL-40 mediate early Alzheimer’s disease progression. Alzheimer's & Dement. 2024;20(1):483–493. 10.1002/alz.13450 PMC10917053 37690071

[41] Jackson TA, MacLullich AMJ, Gladman JRF, Lord JM, Sheehan B. Diagnostic test accuracy of informant-based tools to diagnose dementia in older hospital patients with delirium: A prospective cohort study. Age Ageing. 2016;45(4):505–511. 27076526 10.1093/ageing/afw065

