# Supplementary material for: Increased cerebrospinal fluid YKL-40 concentration in hip fracture patients with delirium
Source: Brain Commun. 2026 Feb 25;8(1):fcag005. doi: 10.1093/braincomms/fcag005 (PMC12932948; doi:10.1093/braincomms/fcag005)
Supplement: fcag005_Supplementary_Data [file fcag005_supplementary_data.pdf]

Supplementary material

Statistical analysis: influential observations

In the adjusted logistic regression models for patients with and without dementia, influential observations were identified by the following statistics: Pearson residual, deviance residual, Pregibon leverage and Pregibon's dbeta. We plotted these statistics against predicted values and the index id, and data points located far from the central distribution were investigated further. No data entry errors were found among these. We then conducted the regression models again while removing each identified observation one at a time, and those with substantial impact on the model estimates were ultimately excluded (one in the group without dementia and two in the group with dementia). The excluded observations corresponded to the most extreme CSF YKL-40 concentrations (1242 ng/mL, 985 ng/mL, 792 ng/mL). All three observations were registered without delirium. One had subsyndromal delirium. In the no dementia group, removing the one observation changed the p-value for CSF YKL-40 from 0.001 to <0.001. In the dementia group, removing the two observations changed the p-value for CSF YKL-40 from 0.012 to 0.189.

Results: CFS YKL-40 and delirium, stratified by dementia status

Supplementary Table 1 Characteristics and CSF YKL-40 concentrations of the participants according to delirium status, stratified by dementia status

	**No dementia (*n*=320)**			**Dementia (*n*=225)**		
	**Delirium (*n*=74)**	**No delirium (n=246)**	***P*-value[a]**	**Delirium (*n*=183)**	**No delirium (*n*=42)**	***P*-value[b]**
**First hip fracture cohort, *n***	**17**	**51**		**52**	**10**	
Age, median (IQR)	85 (81-88)	83 (71-88)	0.114	85 (81-90)	87 (78-91)	0.939
Female, *n* (%)	11 (65)	38 (75)	0.435	36 (71)	9 (90)	0.178
ASA III-IV, *n* (%)	12 (71)	24 (47)	0.092	37 (71)	3 (30)	**0.013**
CSF YKL-40, ng/mL, median (IQR)[c]	175 (127-248)	132 (100-190)	**0.010**	162 (119-206)	198 (129-251)	0.339
**Second hip fracture cohort, *n***	**57**	**195**		**131**	**32**	
Age, median (IQR)	86 (79-90)	76 (69-84)	**<0.001**	86 (81-91)	83(74-87)	**0.003**
Female, *n* (%)	37 (65)	131 (67)	0.750	90 (69)	20 (63)	0.500
ASA III-IV, *n* (%)[d]	35 (61)	67 (34)	**<0.001**	92 (70)	20 (63)	0.360
CSF YKL-40, ng/mL, median (IQR)[c]	243 (175-327)	174 (137-231)	**<0.001**	221 (181-308)	228 (185-311)	0.851

IQR = interquartile range (25th percentile - 75th percentile), ASA = American Society of Anesthesiologists physical status classification.
[a]Delirium versus no delirium for hip fracture patients without dementia. Mann-Whitney U test was used for continuous variables, chi-square test for categorical variables. Bold text denotes a significant p-value (*P*<0.05).
[b]Delirium versus no delirium for hip fracture patients with dementia. Mann-Whitney U test was used for continuous variables, chi-square test for categorical variables. Bold text denotes a significant p-value (*P*<0.05).
[c]CSF YKL-40 concentrations are not directly comparable between cohorts.
[d]ASA score was missing in two participants.
